# Targeting immunometabolism as an anti-inflammatory strategy

**DOI:** 10.1038/s41422-020-0291-z

**Published:** 2020-03-04

**Authors:** Eva M. Pålsson-McDermott, Luke A. J. O’Neill

**Affiliations:** School of Biochemistry and Immunology, Trinity Biomedical Science Institute, Trinity College Dublin, Dublin, Ireland

**Keywords:** Autoimmunity, Innate immunity

## Abstract

The growing field of immunometabolism has taught us how metabolic cellular reactions and processes not only provide a means to generate ATP and biosynthetic precursors, but are also a way of controlling immunity and inflammation. Metabolic reprogramming of immune cells is essential for both inflammatory as well as anti-inflammatory responses. Four anti-inflammatory therapies, DMF, Metformin, Methotrexate and Rapamycin all work by affecting metabolism and/or regulating or mimicking endogenous metabolites with anti-inflammatory effects. Evidence is emerging for the targeting of specific metabolic events as a strategy to limit inflammation in different contexts. Here we discuss these recent developments and speculate on the prospect of targeting immunometabolism in the effort to develop novel anti-inflammatory therapeutics. As accumulating evidence for roles of an intricate and elaborate network of metabolic processes, including lipid, amino acid and nucleotide metabolism provides key focal points for developing new therapies, we here turn our attention to glycolysis and the TCA cycle to provide examples of how metabolic intermediates and enzymes can provide potential novel therapeutic targets.

## Introduction

Immunologists have recently turned their attention to metabolic changes occurring in immune cells that have a determining role in their effector responses.^1^ Pro-inflammatory signals will induce a metabolic switch in both myeloid and lymphoid cells, resulting in upregulation of aerobic glycolysis in a manner similar to the Warburg effect described in cancer cells. This dependence on glycolysis is seen in classically activated M1 macrophages, monocytes, educated NK cells, activated B cells and effector T cells including T-helper (Th) 1 and Th17 cells where glycolysis is required for proper differentiation and immune effector functions. Oxidative phosphorylation on the other hand in general favors an anti-inflammatory phenotype such as that of alternatively activated M2 macrophages and regulatory T cells (T_reg_). A concept is emerging whereby the repolarizing of immune cells towards a less inflamed phenotype by manipulating metabolism using small molecules and metabolic intermediates might be possible. Through the differential metabolic requirements of effector and regulatory immune cell populations we have a therapeutic opportunity that allows for cellular selectivity when regulating immune responses. Targeting metabolic processes therapeutically will, unlike the approach of global immunosuppression, specifically and selectively target cells with high metabolic demands whilst not affecting other immune cells hence potentially reducing unwanted side effects. Prominent examples include reprogramming a classically activated M1 macrophage towards a more anti-inflammatory M2 macrophage using dimethyl fumarate (DMF, a drug currently approved for the treatment of multiple sclerosis), metformin (which is used to treat Type 2 diabetes)^2^ or TEPP-46 which promotes the tetramerization of the key glycolytic enzyme Pyruvate kinase M2 (PKM2). Similarly, repolarization of circulating T cell subsets towards a more anti-inflammatory T_reg_ population in multiple sclerosis has been achieved using dimethyl fumarate (DMF)^3^ and by the immunosuppressive drug rapamycin which suppresses differentiation of pathogenic Th17 cells in favor of immunosuppressive Treg cells.^4^ As we learn more about the importance of rewiring metabolic processes to drive inflammation and disease pathophysiology, novel therapeutic opportunities are likely to be revealed for the treatment of immune disorders. By understanding and defining which specific metabolic requirements fuel a particular inflammatory immune disorder, new therapeutic targets might emerge.

In this review we will discuss small molecules that are currently in use clinically for inflammatory and autoimmune diseases that are likely to act by targeting metabolic processes in immune cells. These disease-modifying immunomodulatory therapies act on metabolic changes including the redox regulatory transcription factor Nrf2, glucose metabolism, one-carbon metabolism and mTOR, a key regulator of glucose, nucleotide, amino acid, fatty acid and lipid metabolism. We will then describe current findings in immunometabolism which might lend themselves to therapeutic targeting, that offer possibilities for whole new approaches for the treatment of multiple diseases involving immune dysfunction. Regulatory, memory and effector immune cell subsets undergo distinct metabolic reprogramming in order that specific nutritional, biosynthetic and energy requirements are met, thereby providing a means of cell specific therapeutic targeting. Intricate metabolic pathways are involved in order to achieve an adequate immune effector response, these include glycolysis, the pentose phosphate pathway, fatty acid metabolism, amino acid metabolism/one-carbon metabolism and the TCA cycle. We will home in on intermediates and enzymes of glycolysis and the TCA cycle and give some specific examples of how anti-inflammatory effects can be achieved by directly impacting on immune cell metabolism.

## Drugs with anti-inflammatory properties that target metabolism

### Dimethyl fumarate

The anti-inflammatory agent DMF is currently used extensively for the treatment of relapse-remitting multiple sclerosis (RRMS) as well as psoriasis and has been shown to alter responses of both innate and adaptive immune cells^5^ (Table 1). Despite the beneficial anti-inflammatory effects of DMF in a wide range of immune cells, including B cells, NK cells, T cells, dendritic cells (DCs) and macrophages, the exact mechanism of how DMF elicits its effects is not fully understood.

**Table 1:** Table summarizing consequences of targeting immune cells with DMF, Metformin, Methotrexate and Rapamycin, listing cell types studied and successful pre-clinical disease models for each.

	Target	Consequence	Immune cells affected	Successful clinical, pre-clinical models
DMF	Keap1/Nrf2 NFkB IRAK4 Cofilin-1/ tubulin/CRMP2 etc	Activation of Nrf2 leading to: Intracellular redox balancing; Regulation of pentose phosphate pathway and fatty acid metabolism Anti-inflammatory effects	T cells B cells NK cells Dendritic cells Monocytes Microglia Neutrophils	RRMS Psoriasis SLE Colitis
Metformin	Inhibition of Complex 1; Activation of AMP kinase	Decreased mitochondrial ROS; Inhibition of IL-1b; Increased FAO; Inhibition of mTORC1	Macrophages T cells B cells	T2D SLE Colitis EAE Obesity
Methotrexate	DHFR (high dose) AICAR (low dose)	Inhibition of nucleotide synthesis; Release of adenosine; Activation of AMPK; Inhibition of pro-inflammatory cytokines	T cells M2 Macrophages Neutrophils Osteoclast formation B cells	RA Crohn’s disease Psoriasis JIA
Rapamycin	mTORC1	Restore cellular homeostasis; Promotes tolerance and generation of memory T cells and tissue-resident macrophages	T cells Macrophages NK cells	RA SLE MS

*RRMS* relapse-remitting multiple sclerosis, *SLE* systemic lupus erythematosus, *T2D* type 2 diabetes, *EAE* experimental autoimmune encephalomyelitis, *RA* rheumatoid arthritis, *JIA* juvenile idiopathic arthritis, *MS* multiple sclerosis, *DHFR* dihydrofolate reductase, *AICAR* amido-imidazole-carbox-amido-ribonucleotide, *mTOR* mammalian target of rapamycin.

The importance of antibody-independent pathogenic roles of B cell antigen presentation and cytokine production has been demonstrated in MS. B cell depletion is an effective cell-directed therapy for MS.^6^ DMF treatment preferentially reduces the population of pro-inflammatory cytokine producing B cells.^7^ Furthermore, DMF promotes cytotoxicity and degranulation of Natural Killer (NK) cells, and by altering populations of NK cells, DMF increases CD56^bright^ NK cell numbers, thereby limiting autoimmunity through controlling the population of autoreactive T cells.^8^ In addition, analyzing subsets of peripheral immune cell subsets from MS patients undergoing DMF therapy revealed an expansion of FoxP3^+^ regulatory T cells, CD56bright NK cells and plasmacytoid dendritic cells accompanied by a decrease in CD8^+^ T cells, B cells and type 1 myeloid dendritic cells.^9^

Limiting peripheral autoreactive T cell counts following treatment with DMF was a phenomenon first identified in psoriasis patients, but has also been reported in MS patients.^10^ This drop in primarily CD8^+^, but also to a lesser extent CD4^+^ T cell numbers, is caused by an increased induction of apoptosis and a reduced rate of proliferation.^11^ Interestingly, a simultaneous increase in naïve CD3^+^CD4^+^ and CD3^+^CD4^−^ T cells was observed in DMF treated patients compared to untreated controls.^11,12^ This likely contributes to the beneficial effects of DMF since decreased numbers of naïve T cells is a causative factor in the development of RRMS. Furthermore, DMF causes a relative increase in anti-inflammatory CCR3^+^ Th2 and Treg cells, with an overall decrease in absolute numbers of T_regs_ and memory T cells, altering the balance between Th1/Th17 and Th2 T cells. Recent data also demonstrate DMF-mediated repression of IL-17-producing CD8^+^ (Tc17) cell frequency in peripheral blood mononuclear cells from MS patients.^13^

The pronounced anti-inflammatory repolarization of T cell populations following treatment with DMF is likely due to changes to the antigen presenting cell populations. Rather than affecting absolute numbers of DCs or monocytes, their polarization and function is altered towards a more anti-inflammatory profile or an M2 phenotype in the case of monocyte-derived macrophages.^11,12^ Monocytes, microglia and DCs from MS patients treated with DMF exhibit a decreased expression of LPS-induced pro-inflammatory cytokines, as well as a reduced expression of the pro-inflammatory micro-RNA miR-155.^14–16^ Apart from reducing the release of IL-1β, TNFα and IL-6, DMF also reduces nitric oxide synthase production in microglial cells, whilst inducing Nrf2 protein expression.^17^ Finally, DMF treatment causes a decrease in the number of CD19^+^ B-lymphocytes in MS patients, coupled to a drop in GM-CSF, IL-6 and TNFα.^7,18,19^

Despite extensive research on the beneficial immunomodulatory effects of DMF and its hydrolyzed counterpart mono methyl fumarate (MMF), the exact mechanism of action for DMF has yet to be determined.

DMF promotes expression of antioxidant genes by stabilizing the ubiquitously and constitutively expressed transcription factor Nuclear Factor (erythroid-derived 2) like 2 (Nrf2 or NFE2L2).^20^ Under resting basal conditions, Nrf2 levels are maintained through ubiquitination and degradation, a process that is regulated by binding of Nrf2 to two molecules of Kelch like-ECH-associated protein-1 (Keap1). When bound to Keap1, Nrf2 can become ubiquitinated by Cullin-3 E3 Ligase, leading to rapid proteosomal degradation.^21^ DMF covalently modifies cysteine residues on Keap1 resulting in conformational changes and release of Nrf2 from the complex. It might therefore mimic the endogenous metabolites fumarate and itaconate, both of which have anti-inflammatory properties and have been shown to target similar cysteines, nicely exemplified by cysteine modifications on Keap1 and GAPDH by itaconate and DMF.^22–24^ The modifications of cysteines on Keap-1 allows existing and de novo synthesized Nrf2 to translocate to the nucleus where it binds the antioxidant responsive elements (ARE) of the promoter region of numerous important genes, helping to maintain redox balance. These include the gene encoding glutamate cysteine ligase, which is responsible for the synthesis of the vital antioxidant glutathione (GSH), and that for glutathione S-transferase (GST), which catalyzes the conjugation of GSH to xenobiotic compounds enabling their detoxification and elimination. Furthermore, Nrf2 promotes the expression of NAD(P)H quinone oxidoreductase 1 (NQO1) responsible for reduction and detoxification of reactive quinones, and the expression of the enzyme responsible for the degradation of heme to biliverdin, Heme oxygenase-1 (HMOX1).^25^ Interestingly, HMOX1 acts, in part, as an antioxidant but has also been shown to be anti-inflammatory by reducing levels of IL-12 p40, IL-16 and TNFα in dendritic cells and stimulating Treg cells during allergic airway inflammation.^26,27^ Several studies have demonstrated beneficial therapeutic effects of DMF in experimental models of colitis. DMF inhibits neutrophil infiltration and release of pro-inflammatory mediators in an inflammatory model using intracolonic administration of dinitrobenzene sulfuric acid (DNBS).^28^ Furthermore, DMF alleviates signs of disease in a model of dextran sulfate sodium (DSS)-induced mouse colitis through a mechanism involving activation of Nrf2, decreased mitochondrial ROS, and suppression of the NLRP3 inflammasome.^29^

The pentose phosphate pathway runs in parallel to glycolysis, oxidizing glucose to generate large quantities of NADPH, a critical cofactor in reducing glutathione disulfide into the antioxidant glutathione. Several studies have reported how Nrf2 regulates the pentose phosphate pathway in cancer cells as well as immune cells.^30,31^ The microRNAs miR-1 and miR-206 regulate gene expression of key enzymes of the pentose phosphate pathway via Nrf2 and HDAC4.^32^ In addition, Nrf2-driven expression of telomerase reverse transcriptase is required for the expression of glucose-6-phosphate dehydrogenase (G6PD) and transketolase of the pentose phosphate pathway in glioblastoma.^33^ In the context of hypercholesterolemia, Nrf2-deficient macrophages displayed downregulated expression of several genes of the pentose phosphate pathway, including 6-phosphogluconate dehydrogenase.^31^ Somewhat surprising therefore, in human erythrocytes, DMF has been shown to inhibit G6PD, the rate-limiting enzyme of the oxidative branch of the pentose phosphate pathway, resulting in modulation of NADPH-dependent glutathione reductase activity, as well as induction of eryptosis and cell shrinkage.^34^

Furthermore, DMF modulates lipid metabolism and fatty acid levels in MS patients, changes that correlate with changes in immunological parameters such as absolute lymphocyte counts and CD8^+^ T cell subsets.^35^ Nrf2 drives mitochondrial fatty acid oxidation,^36^ whilst suppressing both lipid biosynthesis and expression of key desaturation genes including fatty acid synthase (FAS) and stearoyl-CoA desaturase 1.^37^

By regulating the expression of a vast range of genes, Nrf2 aids in xenobiotic responses, intracellular redox balancing, cell survival, and metabolic regulation of the pentose phosphate pathway and fatty acid metabolism. Together, these are likely to contribute to the anti-inflammatory effects of DMF in MS and other inflammatory disorders.

The highly electrophilic DMF reacts, not only with cysteine on Keap1, but covalently modifies a wide range of other protein thiols and has been shown to exert anti-inflammatory effects independently of Nrf2.^38^ In breast cancer cells, DMF was shown to covalently modify the NFκB subunit p65 on cysteine 38, preventing nuclear translocation and DNA binding, which may be beneficial when treating aggressive cancers that rely on NFκB for growth, angiogenesis and therapeutic resistance.^39^ Using mass spectrometry, Piroli et al. recently identified 24 novel DMF-modified proteins in neurons and astrocytes, including cofilin-1, tubulin and collapsing response mediator protein 2 (CRMP2).^40^ Using human plasmacytoid DCs, an important cell type in systemic lupus erythematosus (SLE), as well as in psoriasis and MS, Zaro et al. recently described anti-inflammatory effects of DMF that are dependent on cysteine-13 of IRAK4,^41^ a key enzyme in IL-1, IL-18 and Toll-like receptor (TLR) signaling. Covalent modification at this site by DMF prevents the assembly of IRAK4 and the adapter protein MyD88, leading to a reduced production of cytokines and chemokines including IFN-α and TNFα, thereby identifying cysteine 13 on IRAK4 as a vital regulatory point for adequate innate immune responses and potentially a novel therapeutic target for suppressing an overactive immune system.

DMF and its active metabolite MMF are also agonizts of the G-protein coupled receptor hydroxycarboxylic acid receptor 2 (HCA_2_, also known as niacin receptor 1 and GPR109A), which belongs to the nicotinic acid receptor family.^42^ HCA2 is the receptor for niacin, a form of Vitamin B3 and the precursor of the coenzymes nicotinamide adenine dinucleotide (NAD) and nicotinamide adenine dinucleotide phosphate (NADP).^43^ Niacin is widely used in treating high blood cholesterol and pellagra. However, as niacin binding to HCA_2_ induces Prostaglandin E2, this causes vasodilation and flushing, thereby restricting the lipid lowering therapeutic use of niacin.^44,45^ This consideration also applies to DMF, which similarly causes flushing. HCA_2_ is expressed by keratinocytes and immune cells, particularly by neutrophils and macrophages, and plays an important role in mediating the anti-inflammatory effects of DMF in experimental autoimmune encephalomyelitis (EAE), a mouse model of MS.^42^ DMF lowered the number of spinal inflammatory cells including neutrophils and reduced demyelination with none of the protective effects of DMF evident in *hca2*^−/−^ mice, suggesting that HCA_2_ is required for the therapeutic benefits of DMF.^42^

These complicated changes in peripheral immune cell populations all contribute to the global anti-inflammatory effect of DMF in MS patients.

### Metformin

Metformin is a well-established compound for controlling glucose metabolism (Table 1).^46^ It is a biguanide which is used widely in type 2 diabetes mellitus (T2DM). Persistent insulin resistance and progressive beta cell failure leads to increased plasma glucose concentration (hyperglycemia), with a large inflammatory component central to the pathology and disease progression of T2D. Proinflammatory IL-1β not only induces apoptosis in pancreatic beta cells,^47^ but also contributes to insulin residence via c-Jun N-terminal kinase (JNK).^48^ The causative protein of T2D pancreatic amyloid deposits, islet amyloid polypeptide (IAPP), triggers the Nlrp3 inflammasome and generates mature IL-1β^49^ further pointing to IL-1β as an important inflammatory mediator of T2D.

Metformin is the first line of oral antidiabetic treatment and is used because of its ability to control disturbed glucose metabolism. Following initial treatment, Metformin is usually part of subsequent combination therapies.

Metformin acts by reducing hepatic gluconeogenesis and improving glucose uptake. However recent studies point to anti-inflammatory modes of action of metformin. Metformin inhibits the mitochondrial electron transport chain at Complex I (NADH:ubiquinone oxidoreductase). The first evidence that modulating the electron transport chain can alter the response of inflammatory cells came from the demonstration that metformin inhibits reactive oxygen species (ROS) generated through reverse electron transport at Complex I and consequently inhibits production of IL-1β in LPS-activated macrophages.^2,50^ Furthermore, inhibition of Complex I leads to a reduction of intracellular ATP production which is sensed by AMP-activated protein kinase (AMPK).^51,52^ The ensuing upregulation of AMPK activity impacts on levels of cholesterol and triglyceride and stimulates fatty acid oxidation while inactivating acetyl-CoA carboxylase (ACC), a rate limiting enzyme for fatty acid synthesis. Hence, activation of AMPK by metformin may, at least in part, provide the mechanism for the observed metformin-mediated reduction of visceral fat, and upregulation of enzymes related to fatty acid oxidation.^53^ Although activation of AMPK by Metformin results in decreased overall gluconeogenic gene transcription,^54^ many beneficial effects of metformin are most likely independent of AMPK. Using AMPK-deficient mice, the effects of metformin on hepatic glucose output remained intact, with comparable levels of blood glucose observed in wild type mice as in mice lacking hepatic AMPK, demonstrating inhibition of heptic gluconeogenesis by metformin independently of AMPK.^55^ Furthermore, metformin has been shown to inhibit antigen-induced T cell proliferation and mammalian target of rapamycin complex I (mTORCI) in T cells hence inhibiting expression of the transcription factors c-myc and hypoxia-inducible factor 1 alpha (Hif-1α), all in an AMPK-independent manner.^56^ Metformin also improves impaired B cell function associated with T2D in vivo; improves antibody responses; and reduces B cell-intrinsic inflammation in vitro.^57^

An anti-inflammatory effect has also been shown in models of systemic lupus erythematosus (SLE), colitis, experimental autoimmune arthritis (EAE) and obesity, all of which indicate the potential of Metformin as an anti-inflammatory agent acting via altered metabolism in immune cells.^58–61^

### Methotrexate

In 1948, Farber, discovered how aminopterin (4-aminopteroyl-glutamic acid), a folate analog that inhibits one-carbon transfer reactions required for de novo nucleotide synthesis, caused remission in children with acute myeloid leukemia (AML).^62^ This and other early clinical progress lead to the development of a class of drugs including antifolates, called ‘antimetabolites’, small nucleotide analogs that inhibit enzymes of nucleotide synthesis.^63^ Since it was first introduced, Methotrexate, an antifolate closely related to aminopterin, has been used as a safe and effective chemotherapy treatment against a wide range of cancers including leukemia, lymphoma, breast, head and neck as well as lung cancer. For several decades methotrexate has also been successfully used at low doses to treat autoimmune diseases such as rheumatoid arthritis (RA), Crohn’s disease and psoriasis (Table 1). In RA it is the most commonly used drug due to its efficacy, safety and cost, and it is currently the gold standard mono- or combination therapy, although adverse events and non-effectiveness cause a number of patients to discontinue treatment, with men responding better than women.^64^

At the doses of methotrexate required for efficacy in cancer, it acts as a competitive inhibitor of dihydrofolate reductase (DHFR), hence it will reduce downstream intermediates of the folate pathway, and ultimately inhibit nucleotide synthesis, resulting in impaired DNA replication and repair.^65–67^ This mechanism for efficacy in cancer treatment results in arrest in the S phase of the cell cycle eventually leading to apoptosis. Adverse effects can be reversed with high doses of calcium or folic acid. At lower doses, such as those used when treating inflammatory diseases, the mechanism of action cannot entirely be attributed to DHFR blockade.^68^ The immunosuppressive and anti-proliferative effects observed in RA, Crohn’s disease and psoriasis are obtained when methotrexate is typically administered at 1/100^th^ of the dose used for cancer and the mechanism of action remains somewhat unclear.^69–71^ The efficacy of low-dose methotrexate in the treatment of RA is unaffected by folate supplements, suggesting that the mechanism here is not folate antagonism and inhibition of purine metabolism.^72^ When administered orally, low-dose methotrexate reaches a peak plasma concentration at 1–2 h and is cleared from circulation after 24 h. Methotrexate is actively taken up by the folate transporter 1 (FOLT) enabling its steady intracellular accumulation. A likely mechanism of action of methotrexate at low-doses is through blocking amido-imidazole-carbox-amido-ribonucleotide (AICAR) transformylase, resulting in elevated intracellular levels of AICAR, leading to release of the paracrine signaling molecule adenosine.^68^ AICAR, an activator of AMP kinase and, as such, an imitator of energy deprivation, supresses T cell activation.^73^ Extracellular adenosine then exerts its anti-inflammatory properties via the adenosine A_2A_ receptor, promoting polarization of a differentially activated (M2) macrophage phenotype, inhibiting adhesion and recruitment of neutrophils, blunting osteoclast formation and bone degradation as well as acting inhibitory on T cell activation and proliferation while promoting T_reg_ differentiation (reviewed in^74^ and^75^). Interestingly, immune cells from patients with RA have upregulated surface expression of adenosine receptors, which might make them more susceptible to treatment with methotrexate.^76^ Methotrexate also inhibits production of pro-inflammatory cytokines such as T cell derived IFNγ, IL-4, IL-13 and TNFα in whole blood as well as monocytes from RA patients, most likely due to a decreased Th1 and Th2 population compared to RA patients not undergoing treatment with methotrexate.^77^ B cells play a central role in the pathogenesis of both RA and juvenile idiopathic arthritis (JIA) and methotrexate significantly decreases absolute numbers of CD19^+^ B cells as well as CD27− transitional B cells, coupled to a reduced serum immunoglobulin level in patients with JIA,^78^ which likely contributes to the anti-inflammatory effects of using methotrexate in managing progression of the disease.

Methotrexate may also reduce inflammation by scavenging free radicals, attenuating intracellular oxidative stress, and inhibiting formation of immunogenic proteins complexes called MMA-adducts.^79^ Methotrexate reduces cell proliferation as well as monocyte adhesion, and increases the rate of apoptosis of T cells, all of which is regulated by cellular reactive oxygen species (ROS).^80–82^

Somewhat similar to DMF therefore, methotrexate may leverage an endogenous anti-inflammatory process in the form of adenosine.

### Rapamycin

A central mediator of inflammation and sensor of the metabolic status of the cell is the phosphoinositide 3-kinase (PI3K)/Akt/mechanistic mammalian target of rapamycin (mTOR) pathway. A plethora of evidence presented here and reviewed more in depth elsewhere (ref. ^83^) of the central role for mTOR in inflammation suggest that targeting mTOR with Rapamycin or derivatives thereof may ultimately provide us with a novel therapeutic target for altering the course of immune disorders and inflammation. Akt is the upstream activator of mTOR which, as a catalytic subunit, forms part of the two metabolic checkpoints mTOR complex 1 (mTORC1) and mTOR complex 2 (mTORC 2). The mTOR signaling pathway is evolutionary conserved and designed to sense nutrient supplies, growth factors and pathogen-associated molecular patterns and convey this information in order to mount correct immune responses. Activation of mTORC 1 and 2 will generally promote anabolic responses, resulting in increased protein synthesis, glycolysis and other processes that will support proliferation and survival. Inhibition of mTOR will restore cellular homeostasis and promote processes such as antigen presentation and generation of memory T cell populations. ATP citrate lyase (ACLY) activity is also regulated by IL-4 activation of the Akt-mTORC1 signaling pathway, thereby altering histone acetylation and expression of a subset of M2 macrophage-associated genes.^84^ Disruption of the mTORC2 signaling complex by deletion of Rictor inhibits generation of M2 macrophages whereas M1 differentiation remains unaffected, demonstrating a critical role for mTORC2 signaling in M2 macrophage differentiation.^85^ Inhibition of mTORC2 promotes generation of tissue-resident macrophages via induction of FOXO1 and GATA6.^86^ Apart from playing a central role in sensing and integrating environmental cues in order to regulate macrophage effector function, mTOR also modulates T_reg_ cell differentiation, activation and function. Inhibition of mTOR during T cell activation favored the generation of a specific subset of memory-like T_reg_ cells with a similar metabolic phenotype to CD8^+^ memory cells.^87^ Apart from changes in glycolysis and increased protein synthesis, cell metabolic reprogramming involves a marked upregulation of amino acid uptake. Deficiencies in amino acid transporters such as the glutamine transporter Slc1a5 impairs differentiation of Th1 and Th17 cells in an mTORC1-dependent manner, hence mTOR acts as an environmental sensor of intracellular concentrations of glutamine.^88^ Similarly, mTOR conveys information about leucine availability; leucine is a well-known activator of mTORC1 and is essential for T cell activation and proliferation.^89^ Consequently, the leucine antagonist N-acetyl-leucine amide (NALA) inhibits this pathway demonstrating how metabolic inhibition can lead to impaired T cell function.^73,90^

Inhibition of mTOR with Rapamycin promotes tolerance, such that T cells stimulated in the presence of Rapamycin fail to produce IL-2 or IFNγ upon re-challenge, making inhibition of mTOR an attractive approach for preventing transplant rejection.^91,92^ Paradoxically, although administration of high doses of rapamycin attenuates autoimmune disease and can be used to treat transplant rejection, low dose rapamycin appears to enhance memory T cell population and function in inflammatory eye disease.^93^

NK cells also undergo dramatic metabolic reprogramming, a process that is strictly regulated by mTORC1 activity.^94^ Not only is mTORC1 required in order to maintain and attain an highly glycolytic metabolic state, mTORC1 activity is also essential for production of key NK cell effector molecules including IFNγ and granzyme B. The role of NK cells in autoimmune disease as evaluated in various animal models has proven somewhat contradictory with both disease-controlling and disease-promoting outcomes. Whilst involved in the onset and progression of disease through NK cell autoreactivity as well as augmenting inflammation through DC, macrophage and T cell interactions, NK cells have also been implicated to play important roles in successful biotherapies targeting human autoimmune disease.^95^ A correlation between NK cell counts and autoimmune disorders has been reported for RA, SLE, MS and others (reviewed in ref. ^96^).

Activation of mTORC1 precedes the onset of SLE and associated co-morbidities. Sirolimus (Rapamycin) given to patients with clinically active SLE over a period of 12 months in a phase 1/2 trial showed a progressive improvement in disease activity, further supporting the promise of targeting this pathway clinically in inflammatory diseases.^97^

DMF, Metformin, Methotrexate and Rapamycin all suggest that the targeting of metabolic processes might be therapeutically beneficial for inflammatory diseases (Table 1). Part of the mechanism of action of DMF, Metformin and Methotrexate may well be to mimic or promote endogenous anti-inflammatory processes or metabolites, thereby leveraging natural mechanisms to limit inflammation. We will now discuss targets in metabolism that could be useful for the development of novel anti-inflammatory agents.

## Targeting glycolysis with small molecules to elicit an anti-inflammatory effect

A key common feature of many activated proinflammatory immune cells is an upregulated rate of aerobic glycolysis and an increased appetite for glucose. Hence limiting the rate of glycolysis provides an opportunity to shift the metabolic flux specific to the population of immune cells that drives the pathology of a given inflammatory disease. We will here discuss some key examples illustrating strategies aimed at targeting regulatory glycolytic enzymes and nutrient transporters involved in this pathway.

### Hexokinase

Warburg first reported on the metabolic shift observed in cancer cells which have a high glucose dependency and increased rate of glycolysis leading to the majority of glucose being metabolized into lactate, even in the presence of oxygen.^98^ We have since learned that this phenomenon, named the ‘Warburg effect’, also occurs in activated immune cells such as M1 macrophages, B cells, NK cells, DCs and Th17 cells,^99,100^ making it an attractive therapeutic target in cancer as well as inflammatory disease.

**Fig. 1:**
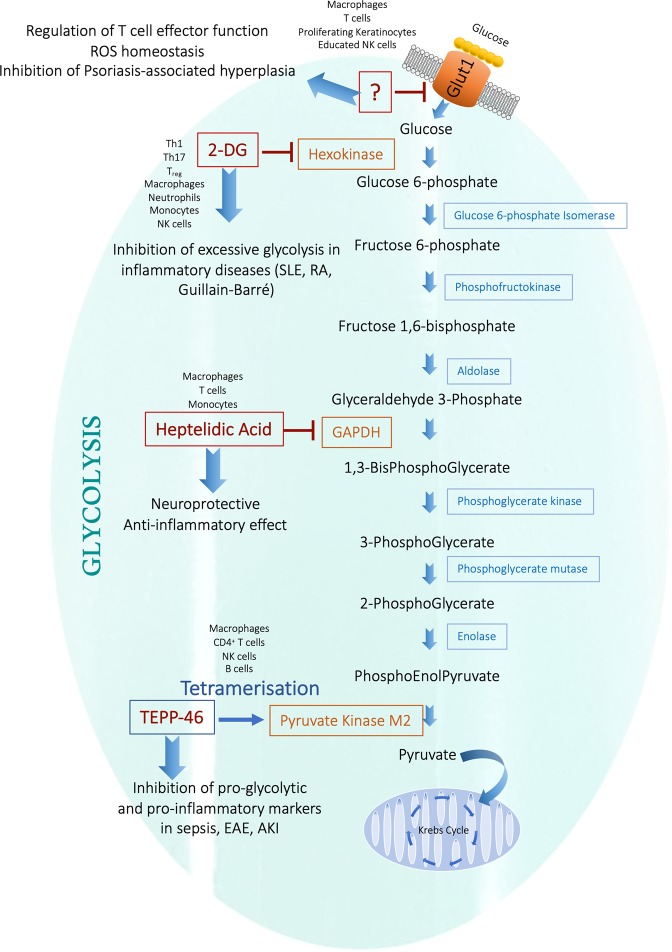
Targeting enzymes of glycolysis as an anti-inflammatory strategy. Inhibition of hexokinase by 2-DG and GAPDH by Heptelidic acid as well as tetramerization and activation of the pyruvate kinase activity of PKM2 by TEPP-46 has proven anti-inflammatory in several models of infection and inflammation.^136^ Targeting the glucose transporter Glut1 may also hold promise as a novel therapeutic target to limit inflammation. Glut-1 glucose transporter-1, 2-DG 2-deoxyglucose, SLE systemic lupus erythematosus, RA rheumatoid arthritis, GAPDH Glyceraldehyde 3-phosphate dehydrogenase, EAE experimental autoimmune encephalomyelitis, AKI acute kidney injury.

One well-described inhibitor of glycolysis is 2-Deoxy-D-glucose (2DG), a glucose analog that blocks glycolysis by acting as a competitive inhibitor to glucose binding to hexokinase in the second step of glycolysis (Fig. 1). 2DG is phosphorylated by hexokinase, resulting in 2DG-6-phosphate which, in turn, will only undergo the first step of the pentose phosphate pathway, leading to accumulation of 2DG-6-phophogluconolactone. Often used as a non-invasive diagnostic tool, [^18^F]-fluoro-deoxyglucose positron-emission-tomography (FDG-PET) is therefore used to visualize glucose uptake in metabolically active lesions but also to identify areas of active inflammation. FDG accumulates in activated macrophages and neutrophils and increased FDG-uptake is associated with lungs and lymph nodes of persons suffering from both active and latent tuberculosis (TB).^101–104^ Using FDG-PET as a diagnostic tool for identifying inflammation and infection is a developing area which now includes detecting granulomatous disease, fungal infection, classification of sarcoidosis, assessment of myocardial viability, as well as evaluating and diagnosing systemic vasculitis, inflammatory bowel disease and active RA.^105^

Outside its application in diagnosis, 2DG may on its own, or in combination therapies, prove useful in inhibiting excessive aerobic glycolysis in inflammatory disorders or malignancies. Inhibition of hexokinase using 2DG impacts on the effector function of most immune cells. 2DG promotes the development of naïve T cells into T_reg_ cells while suppressing Th17 polarization and production of IL-17.^106^ However, the importance of hexokinase for T_reg_ cell immunity has since been questioned.^107^ B cells also rely on hexokinase in order to proliferate and exert apt effector functions, as demonstrated when low doses of 2-DG impaired LPS-induced secretion of IgG and IgM, proliferation, extracellular acidification rate (ECAR) and antibody production, potentially providing a means to promote tolerance in autoimmune and inflammatory disease.^108^ The effector function and reactive potential of NK cells is determined through a process called ‘education’, resulting in variable sensitivity and reactivity.^109^ Glycolysis plays a role for the effector function of educated NK cells such that inhibition of hexokinase and hence glycolysis with 2-DG partially reduced the functional output of killer-cell immunoglobulin-like receptor (KIR)-educated NK cells, however simultaneous inhibition of glycolysis and oxidative phosphorylation is required in order to fully block the cytotoxicity of educated NK cells.^110,111^ Furthermore, activated DCs also rely heavily on glycolysis for their effector functions. 2-DG inhibits expression of co-stimulatory molecule and type I Interferon in Influenza A-stimulated pDCs and impairs migration of GM-DCs as well as splenic CD11c^+^ cDCs.^112,113^

CD4^+^ T cells of SLE rely heavily on glycolysis for their inflammatory effector functions.^58^ 2DG in combination with metformin in vivo normalized T cell metabolism and reduced disease markers such as anti-dsDNA IgG and anti-nuclear autoantibodies (ANA) as well as immune complex deposition in a mouse model using lupus-prone TC mice.^58^ Furthermore, inhibition of glycolysis with 2-DG in the spontaneous arthritis K/BxN mouse model significantly reduced joint inflammation, CD4^+^ follicular helper T cells and Th17 cell expansion as well as frequency of neutrophils and monocytes in the joint draining lymph node.^114^ In contrast to SLE, disease markers in this model of arthritis were not sensitive to metformin, suggesting a critical role for glycolysis in disease progression. Guillain-Barré syndrome, an acute peripheral neuropathy disease driven largely by inflammatory glycolytic macrophages as well as Th1 and Th17 cells, can be modeled in vivo using experimental autoimmune neuritis (EAN) in rats. Daily injections of 2-DG in this model inhibited the initiation of EAN, accompanied by decreased numbers of Th1 and Th17 cells, whilst upregulating the T_reg_ cell population. Furthermore, when dosing therapeutically at a later stage of the disease progression, 2-DG dramatically suppressed EAN progression and severity.^115^ These models show great therapeutic promise for inhibiting glycolysis in inflammatory disorders. Clinically, phase I trials suggest that 2DG is well tolerated at low doses, and favorable early clinical outcomes in cancer patients have been reported.^116–118^

### Glut-1

Many pro-inflammatory immune cells rely on a high rate of aerobic glycolysis to meet the metabolic and biosynthetic demands required to raise an adequate immune response. Part of the mechanism of controlling the rate of glycolysis is by the ability to import glucose, something which is regulated through the expression of the Glut (SLC2A) family of membrane transporters. Of the fourteen transporters expressed in humans, Glut 1, 2, 3 and 4 are the best characterized, with Glut1 expression being closely linked to glycolytically active cells (reviewed in refs. ^119,120^) (Fig. 1).

Part of the mechanism of the pro-glycolytic shift in metabolism in proinflammatory macrophages, is the expression Glut1 (SLC2A1).^121,122^ Glut1 is upregulated on LPS-stimulated macrophages accompanied by an increased rate of glucose uptake.^122^ Furthermore, glucose uptake and expression of Glut1 is one of the rate-limiting steps in achieving an increased cellular uptake of glucose, leading to elevated levels of intermediates of the pentose phosphate pathway, as well as a reduced rate of oxidative phosphorylation. Moreover, macrophages overexpressing Glut1 displayed an increased production of ROS and elevated oxidative stress.^121^ Macrophages are not the only cell type where Glut1 expression has been linked to phenotypic changes. For example, Glut1 promotes T cell effector function, while fatty acid oxidation favors regulatory CD4^+^ T cells and CD8^+^ memory T cell function.^123,124^ Similarly, glycolysis in endothelial cells promotes cell migration and is required for proper vessel formation and branching, whereas fatty acid oxidation is linked to endothelial cell vascular sprouting and de novo synthesis for DNA replication.^125,126^ Blocking uptake of glucose using the glucose-transporter inhibitor CG-5, inhibits Th1 and Th17 differentiation, and promotes T_reg_ polarization whilst ameliorating disease symptoms and reducing expansion of germinal center B cells and production of autoantibodies in a spontaneous lupus-prone mouse model.^127^ Furthermore, Glut1 is critical for the elevated antibody production and increased rate of glycolysis associated with chronic stimulation of B-cell activating factor (BAFF) of B cells in vivo.^108^ Expression of surface Glut1 is also upregulated in KIR-educated NK cells compared to uneducated NK cells.^110^ CD56(bright) NK cells express high levels of Glut1 compared to CD56(dim), are overall more metabolically active and limiting the rate of glycolysis results in impaired production of IFN-γ.^128^ A better understanding of such metabolic differences in subsets of lymphocytic populations and how these affect immune function is important therapeutically for ex vivo culture of immunocompetent immune cells when developing immunotherapy.

Keratinocytes rely largely on Glut1 for glucose uptake, and upon activation such as wound healing, psoriasis or UV-induced hyperplasia, Glut1 expression is further induced. Interestingly Zhang et al. recently demonstrated using *Glut1* deficient keratinocytes, that high reliance on Glut1 and glucose metabolism is uniquely required for proliferating keratinocytes in vivo as would occur in psoriasis.^129^ Whilst *Glut1−/−* primary epidermal keratinocytes displayed impaired proliferation in vitro, surprisingly skin development in these mice in vivo was normal, suggesting Glut1 may be dispensable for normal epidermal development and differentiation. In contrast, Glut1 is critical in psoriasis associated keratinocyte hyperproliferation, or hyperplasia, as evidenced by Glut1-deficient mice being protected from imiquimod-induced psoriasiform hyperplasia compared to wild type mice. Hence, glucose uptake via Glut1 is required for proliferating keratinocytes but not for normal skin development and function. Metabolic reprogramming may allow normal keratinocytes to adapt and survive in the absence of glucose metabolism (or if Glut1 were inhibited), specifically through upregulation of the pentose phosphate pathway and alterations in amino acid metabolism so as to compensate for a limited rate of glycolysis, TCA cycle and nucleotide synthesis. This selective requirement for glucose metabolism for rapidly proliferating keratinocytes may provide a novel target for pathologies involving hyperproliferation of keratinocytes or indeed other cells. Zhang et al. also demonstrated that a Glut1 inhibitor, WZB117,^130^ could block inflammatory gene expression in skin biopsies from psoriasis patients. This study makes Glut1 an attractive therapeutic target in psoriasis.

### PKM2

There are four pyruvate kinase (PK) isoforms, each with a distinct and unique tissue expression pattern. In glycolysis, pyruvate kinase is the rate limiting enzyme responsible for converting phosphoenolpyruvic acid to pyruvate, a reaction that in normal differentiated tissue is mediated by the enzymatically active isoform PKM1.^131^ Multiple studies have reported a key role for pyruvate kinase M2 in immune effector function. The gene that encodes isoforms PKM1 and PKM2 is located on chromosome 15, where the mature RNA produced will be determined as a result of alternative splicing of exons 9 (included in mature *PKM1* mRNA) and exon 10 (included in mature *PKM2* mRNA). This event is highly regulated and in order to obtain mature *PKM2* mRNA, both repression of exon 9, an event regulated by splicing factors PTB, hnRNPA1 and hnRNPA2, as well as the inclusion of exon 10 by SRSF3 are required.^132–135^ PKM1 is expressed in most normal differentiated tissues as well as in resting inactive immune cells. PKM2 on the other hand is upregulated in activated immune cells such as LPS-activated macrophages and CD3/CD28 activated T cells.^136^ A switch from PKM1 to PKM2 expression occurs in some tumor types, but more importantly, PKM2 expression is upregulated in all cancer types investigated.^137,138^ In addition, replacing PKM2 with PKM1 has been found to inhibit tumor proliferation and aerobic glycolysis.^138^

The second pyruvate kinase gene *PKLR* is located on chromosome 1 and tissue specific alternative promoters will give rise to the PKR isoform which is expressed exclusively in erythrocytes, or the slightly shorter PKL, lacking exon 1, which is expressed predominantly in the liver, but also in the kidneys and intestine.

The tertiary structure and assembly of pyruvate kinase is critical for the regulation of its enzymatic activity. PKM2 naturally occurs as a dimer/monomer equilibrium, whereas PKM1 differs from the other isoforms in that it exists as a stable homo tetramer with a high constitutive pyruvate kinase enzyme activity, rendering it insensitive to the allosteric activators that affect the activity of PKM2, PKR and PKL.

Some glycolytic enzymes ‘moonlight’ by carrying out functions separate to their glycolytic activity. Examples include hexokinase which together with mTORC1 play a critical role for nod-like receptor family pyrin domain containing 3 (NLRP3) inflammasome assembly.^139^ GAPDH, as discussed below, can bind to AU-rich RNA sequences in place of NAD^+^ in its Rossman fold, thereby regulating expression of several inflammatory mRNAs. This moonlighting phenomenon also holds true for PKM2 which, in addition to its role as a pyruvate kinase catalyzing the last step of glycolysis, also carries out diverse non-canonical nuclear functions but only in its monomeric or dimeric form (Fig. 1). These include acting as a nuclear protein kinase phosphorylating STAT3 on tyrosine 107 and histone H3 on threonine 11, as well as a transcriptional coactivator of the transcription factor Hif-1α.^140–142^ Phosphorylation of PKM2 on Ser37 leads to recruitment of peptidyl-prolyl cis-trans isomerase NIMA-interacting 1 (PIN1), which in turn aids binding of PKM2 to importin α5 thereby facilitating nuclear translocation.^143,144^ The subcellular localization of PKM2 to the nucleus is further regulated by a multitude of modifications. Events like acetylation of PKM2 by p300 acetyltransferase, sumoylation by SUMO-E3 ligase, and binding of PKM2 to Jumonji C domain-containing dioxygenase 5 (JMJD5) promote nuclear translocation, whereas deacetylation of PKM2 at lysine 433 by Sirt 6 and tetramerization by the glycolytic intermediate fructose 1,6-bis-phosphate and small molecules such as TEPP-46 and DASA-58 all prevent nuclear translocation.^136,145–149^

PKM2 has been shown to play a central role in cancer biology; facilitating cancer cell proliferation, lactate production, as well as DNA and lipid synthesis.^150^ Importantly, PKM2 also regulates immune responses, driving aerobic glycolysis and the expression of pro-inflammatory cytokines in LPS-activated macrophages.^136^ The pyruvate kinase enzymatic activity of PKM2 can be allosterically triggered by forcing the assembly of a tetramer. This occurs endogenously when the metabolites fructose-1,6-bisphosphate, as well as serine and succinylaminoimidazolecarboxamide ribose 5’-phosphate (SAICAR) bind to PKM2.^151^ PKM2 can also be activated using highly specific small-molecule activators such as TEPP-46.^152^ TEPP-46 inhibits inflammatory markers in LPS-induced endotoxemia. Moreover, PKM2 plays a central role in CD4^+^ T cell biology and pathogenicity, accordingly, tetramerization of PKM2 ameliorates experimental autoimmune encephalitis (EAE).^153^ A recent study demonstrates a central role for PKM2 in glycolytic rate and differentiation of Th1 and Th17 cells. PKM2 interacts directly with calcium/calmodulin-dependent protein kinase IV (CaMK4) which promotes the activity of PKM2. Furthermore, silencing *Pkm2* limited clinical score in EAE.^154^

The process whereby the inhibitory receptors of an NK cell interacts with self MHC-I molecules allows for the development of fully mature NK cells, and is termed NK licensing, or classic NK cell education. Interestingly, unlicensed NK cells depend entirely on mitochondrial respiration for cytolytic function. Licensed KIR^+^ NK cells isolated from human peripheral blood on the other hand, undergo a metabolic switch with an upregulated rate of glycolysis and mitochondrial-dependent glutaminolysis and in this process will rely on the upregulated expression of PKM2.^111^ Evidence for a role for PKM2 in B cell mediated pathology comes from a model of hyperhomocysteinemia (HHcy)-accelerated atherosclerosis where homocysteine activated B cells display an increase in glycolysis, coupled to an upregulation of PKM2.^155^ Shikonin, an inhibitor of PKM2, restored homocysteine-induced B cell proliferation as well as antibody secretion both in vitro and in vivo, and reduced HHcy-accelerated atherosclerotic lesion formation.

The fact that the kinase activity, as well as cellular localization and nuclear functions of PKM2, can be regulated by small molecule allosteric activators, taken together with the pleotropic biological functions of this enzyme, indicates that PKM2 might be an attractive target not only for tumor therapy, but also when targeting an overactive immune system.^136^ Recent evidence points to a key role for PKM2 in the pathogenicity of acute kidney injury (AKI).^156^ Zhou et al. report a novel regulatory mechanism of PKM2 activity, namely S-nitrosylation. PKM2 of renal proximal tubules is regulated by the S-nitroso-CoA (SNO)/S-nitroso-CoA reductase (SCoR) system whereby oxidative modification of Cys on PKM2 forms S-nitrosothiol (SNO) and provides a means of sensing eNOS activity and protecting against renal kidney injury. Increased S-nitrosylation of renal PKM2 decreased PKM2 tetramerization and activity, forcing glucose flux through the pentose phosphate pathway to detoxify ROS and may also aid in regenerating tissue following injury such as AKI.

An example for some promising outcomes of targeting PKM2 pre-clinically is illustrated by the finding that tetramerization of PKM2 corrects the proinflammatory phenotype of macrophages from patients with coronary artery disease.^157^ Furthermore, increased expression of intestinal PKM2 in mice with TNBS-induced colitis as well as in stool samples from patients with active Crohn’s disease, together with elevated levels of PKM2 in synovial tissue from patients with rheumatoid arthritis all support an unfavorable role for PKM2 in multiple inflammatory disorders, making it an attractive target therapeutically.

### GAPDH

Glyceraldehyde 3-phosphate dehydrogenase (GAPDH) is the enzyme that catalyzes the sixth step of glycolysis, a two-step conversion of glyceraldehyde 3-phosphate into 1,3-bisphosphoglycerate (Fig. 1). Although in general terms the three rate limiting/rate controlling enzymes of glycolysis are hexokinase, phosphofructokinase, and pyruvate kinase, a recent study proposes GAPDH as a rate limiting enzyme during Warburg metabolism.^158^ Using metabolic control analysis, GAPDH was identified as the enzyme with the most prominent difference in regulatory properties during Warburg metabolism, compared to tissues and cell types not undergoing Warburg metabolism. Hence partial inhibition of GAPDH with heptelidic acid, also known as koningic acid, may provide a means of selective toxicity towards tumors or glycolytic active immune cells whilst being well tolerated by other healthy tissues.^159^

Similar to PKM2, GAPDH has been shown to play roles outside glycolysis. GAPDH regulates transcription by binding to DNA, moving between the cytosol and nucleus of the cell. Once GAPDH is S-nitrosylated by NO, translocation of GAPDH together with the E3 ubiquitin ligase SIAH1 to the nucleus results in initiation of apoptosis. Deprenyl, also known as Selegiline, has been used successfully to ameliorate progression in early Parkinson’s disease. Although it functions as a selective, irreversible inhibitor of monoamine oxidase, Deprenyl has been shown to exert its neuroprotective actions by preventing S-nitrolsylation, as well as nuclear translocation of GAPDH/SIAH1 at subnanomolar concentrations.^160^ Similar observations have been made using the monoamine oxidase inhibitors TCH346 and Rasagiline, both of which prevent nuclear translocation of GAPDH, thereby acting in a neuroprotective manner.^161–163^

Another key role of GAPDH involves binding to AU-rich elements in TNFα as well as IFNγ mRNA transcripts.^164,165^ In the case of TNF, GAPDH binds to the 3′ untranslated region of TNF mRNA, leading to post-transcriptional repression in galactose-fed monocytes with limited availability of glucose and hence reduced rate of glycolysis. This repression of TNF translation can be reversed by increasing glycolysis at which time GAPDH binding to mRNA is lowered, thereby providing a means of fine-tuning the inflammatory response.^164^ Chang et al. demonstrated that although activated T cells utilize aerobic glycolysis, and this pathway is critical for T cell effector function, it is not required for proliferation or survival. IFNγ expression in activated T cells is regulated by the binding of GAPDH to AU-rich elements within the 3′ UTR of IFNγ mRNA in a similar manner to the regulation of TNFα expression in monocytes.^165^ In LPS-activated macrophages an upregulation of the citrate-derived metabolite malonyl-CoA will be used as a substrate for the post-translational modification malonylation. Malonylation of GAPDH will alter the ability of GAPDH to bind to mRNA. In resting macrophages, GAPDH preferentially binds to and suppresses TNF mRNA. However, upon cell activation, malonylation of GAPDH will cause dissociation from TNF mRNA thereby promoting inflammation by increased expression of pro-inflammatory TNFα and also by simultaneously allowing GAPDH to engage in glycolysis. Hence, targeting GAPDH therapeutically may prove clinically beneficial. In fact, recent data suggests that the beneficial effects of DMF in multiple sclerosis comes from DMF targeting, succinating and inactivating the catalytic cysteine within the active site of GAPDH.^24^ This provides a proof-of-concept in patients that GAPDH might be an intriguing target for an anti-inflammatory effect.

## Modulating the TCA cycle therapeutically to limit inflammation

During conditions where oxygen is readily available, pyruvate is generated through glycolysis, a ten-step break down reaction of glucose. Pyruvate is then oxidized through a series of reactions termed Kreb’s cycle or the tricarboxylic acid (TCA) cycle. During periods of low oxygen supply or high energy demand pyruvate can instead be converted to lactate by lactate dehydrogenase (LDH). Recent data demonstrate that circulating lactate can be used as the primary source of carbon for the TCA cycle in all tissues except for brain.^166^

**Fig. 2:**
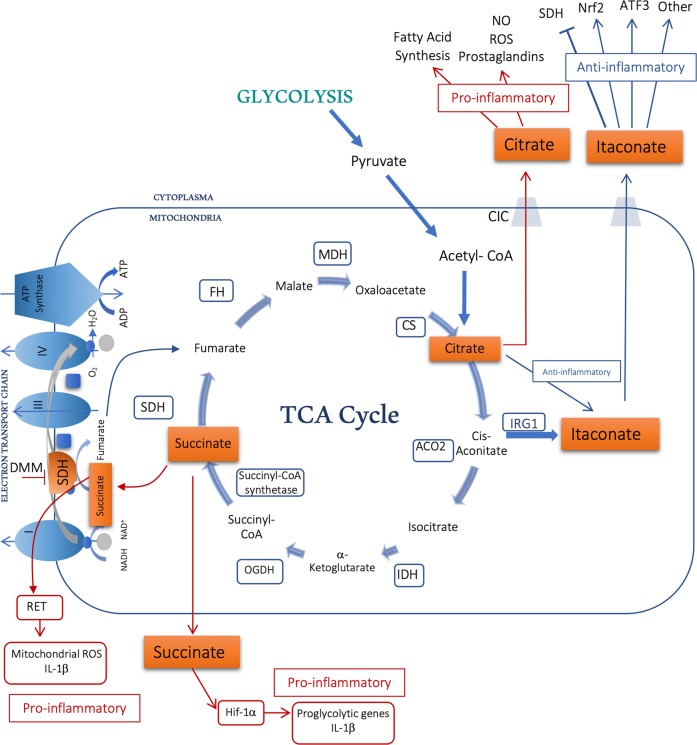
Modifying intermediates of the TCA cycle to drive an anti-inflammatory response. Located in the mitochondrial matrix, the TCA cycle serves to break down nutrients such as glycolysis-generated pyruvate. Metabolic intermediates such as succinate, itaconate and citrate all play a role in inflammation. Enzymes include citrate synthase (CS), isocitrate dehydrogenase (IDH), aconitase (ACO2), α-ketoglutarate dehydrogenase (OGDH), succinyl-CoA synthestase, succinate dehydrogenase (SDH) which makes up Complex II of the electron transport chain, fumarase (FH), malate dehydrogenase (MDH) and Immuneresponsive gene-1 (IRG-1), also known as Aconitate Decarboxylase 1 (ACOD1), the enzyme responsible for the production of itaconate.

The TCA cycle and the succeeding electron transport chain provide a highly efficient means of generating ATP, however key metabolic intermediates serve not only as precursors in biosynthetic pathways but have now also been linked to regulating distinct cellular effector functions. This phenomenon is not unique to the TCA cycle. Intracellular as well as exogenous metabolites such as lactate and fatty acids can be sensed by immune cells and lead to modification of metabolism and effector functions. We will here describe three TCA cycle metabolites and give some brief examples of how modulating these can rework immune responses, thereby illustrating how we are presented with novel potential opportunities for targeting immunometabolism therapeutically (Fig. 2).

### Succinate/DMM

Succinate is arguably one of the most important metabolites, serving at the crossroads of multiple metabolic pathways. After being generated in the mitochondria from succinyl-CoA by the enzyme succinyl-CoA synthetase, succinate can leave the mitochondrial matrix and function in the cytosol as well as extracellularly (Fig. 2). Succinate can be further oxidized into fumarate by respiratory complex II of the electron transport chain, also known as succinate dehydrogenase (SDH). Here succinate serves as a direct electron donor.

First described as an oncometabolite, succinate was identified as a driver of the Warburg effect by inhibition of prolyl hydroxylases leading to reduced hydroxylation and hence stabilization and activation of the transcription factor Hif-1α.^167^ The central role of succinate in immune cell function first came from the observation that accumulation of succinate in LPS-activated macrophages resulted in activation of Hif-1α and subsequent upregulation of the pro-inflammatory cytokine IL-1β.^168^ Hif-1α is a key mediator in mounting both innate and adaptive immune responses, and a transcription factor that senses and responds to the metabolic state of the cell. During normoxic conditions, Hif-1α protein levels are controlled through continuous degradation via the von Hippel-Lindau tumor suppressor protein (VHL)-mediated ubiquitin protease pathway, an event promoted by hydroxylation of the oxygen dependent degradation domains (ODDs) of Hif-1α by prolyl hydroxylases (PHD) (reviewed in ref. ^169^). LPS-activated M1 macrophages have an increased rate of aerobic glycolysis and a perturbed TCA cycle, resulting in an intracellular accumulation of succinate.^168^ Inhibition of prolyl hydroxylase (PHD) by succinate causes reduced hydroxylation and degradation of Hif-1α, resulting in an increase in Hif-1α which will further support glycolysis though promoting expression of pro-glycolytic genes such as hexokinase 1. Hence, Hif-1α is a key contributor to the metabolic reprogramming that occurs in activated macrophages. Whilst aiming to limit Hif-1α expression may be a prime strategy for anticancer therapies and controlling excessive inflammation, target genes of Hif-1α play a critical role in angiogenesis, cell proliferation/survival and glucose/iron metabolism presenting therapeutic opportunities in stabilizing Hif-1α when treating anemia associated with chronic kidney disease or hypoxia-ischemia during myocardial infarction, stroke and perinatal asphyxia.^170–173^ Dimethyloxalylglycine (DMOG) is a potent driver of Hif-1α activity and acts through the inhibition of PHD resulting in stabilization of Hif-1α protein expression and expression of Hif-1α target genes such as vascular endothelial growth factor (VEGF) and proglycolytic enzymes.^174^ Hif-1α plays a key role in promoting expression of erythropoietin (EPO) thereby stimulating production of hemoglobin and red blood cells. Several PHD inhibitors are currently in clinical trials for the treatment of renal anemia and ischemic disease.^175,176^ PHD inhibitors such as Vadadustat, Daprodustat, Molidustat and Roxadustat are all in phase 3 clinical trials for patients with renal anemia secondary to chronic kidney disease.

Conversely, PHD activity can be boosted using a derivative of α-ketoglutarate, leading to depletion of Hif-1α, and a reduction of inflammatory markers in LPS-activated macrophages.^168^

Succinate has been shown to accumulate and play a detrimental role in regulating ischemia-reperfusion (IR) injury^177^ by driving mitochondrial generation of ROS. Purine nucleotide breakdown during ischemia gives rise to fumarate which in turn will drive the reverse enzymatic reaction of SDH, consequently giving rise to accumulation of succinate. Once reperfusion occurs this vast source of succinate will drive the electron transport chain in reverse, causing excessive ROS production at complex 1. Interestingly, the same phenomenon occurs in LPS-activated macrophages, where elevated succinate levels drives the reverse electron transport chain and gives rise to elevated mitochondrial ROS production and Hif-1α dependent induction of pro-inflammatory genes such as IL-1β.^178^ Inhibition of SDH using dimethyl malonate (DMM), a precursor of the competitive SDH inhibitor malonate, favors an anti-inflammatory phenotype and boosts the production of several anti-inflammatory cytokines, including IL-1RA and IL-10. DMM, when administered intravenously, protected against cardiac IR injury in vivo.^177^ Inhibition of SDH by DMM has also been shown to alleviate brain injury following cardiac arrest, resulting in reduced neuronal apoptosis, inhibition of caspase-3 activation and reduced generation of mitochondrial ROS.^179^ Furthermore, DMM proved anti-inflammatory in an LPS/D-galactosamine induced liver injury model, where inhibition of SDH resulted in decreased plasma IL-6 and TNFα levels and improved survival.^180^

During conditions such as rheumatoid arthritis, cold exposure or ischemia reperfusion injury, succinate is released outside of the cell.^181–183^ When the succinate receptor was first identified as a previously orphan G-protein coupled receptor (GPCR) SUCNR1 (formerly known as GPR91),^184^ this landmark study proposed that succinate in the extracellular space can act as a ligand and may have signaling properties beyond a traditional role in metabolism. SUCNR1 has been described as a metabolic sensor and, as a member of the rhodopsin-like GPCR family, consequently making it a very attractive drug target. Several succinate receptor ligands have been described, including the succinate receptor agonizts *cis*-epoxysuccinic acid (*c*ESA) and *cis*-1,2-cyclopropanedicarboxylic acid (*c*CPDA) which both demonstrate an efficacy similar to that of endogenous succinate.^185^ Binding of succinate to SUCNR1 causes cardiac hypertrophy through the ERK1/2 signaling pathway, with detectable elevated serum succinate levels in patients with cardiac hypertrophy.^182^ Succinate also has a synergistic effect on TLR signaling, boosting pro-inflammatory cytokines as well as increasing the antigen presenting ability of dendritic cells.^186^ Furthermore, synovial fluid from rheumatoid arthritis patients with high levels of succinate exhibits a SUCNR1-dependent increase in the expression of IL-1β, providing more evidence of inhibition of SUCNR1 as a potentially interesting anti-inflammatory target.^183^

### Itaconate

The recently described immunomodulatory derivative of the TCA cycle, itaconate, has been particularly well studied in macrophage function where a broad range of anti-inflammatory mechanisms have been described. Itaconate is generated through decarboxylation of cis-aconitate by immunoresponsive gene 1 (IRG-1) in the mitochondrial matrix (Fig. 2). Although initially described as having antimicrobial properties, we now know that itaconate inhibits pro-inflammatory markers in classically activated macrophages. Mechanistically, itaconate has been shown to exert its anti-inflammatory actions through a broad range of mechanisms. The itaconate analog 4-OI activates the anti-oxidant transcription factor Nrf2 through 2,3-dicarboxypropylation of Keap-1 in a manner similar to DMF. Itaconate has been shown to inhibit succinate dehydrogenase (SDH) and thereby block IL-1β production similar to DMM, and regulate IkBζ and IL-6 expression via the transcription factor ATF3.^23,187–189^ The ability of itaconate to influence key immunomodulators including Nrf2 and ATF3 makes boosting cellular itaconate levels an attractive proposition therapeutically. The itaconate mimic 4-octylitaconate (4OI) was protective in a mouse model of septicemia, with increased survival and reduced expression of pro-inflammatory cytokines.^23^ Moreover, the cell penetrable methyl ester derivative of itaconate, dimethyl itaconate (DI), was shown to ameliorate IL-17-IkBζ-driven skin pathology in a mouse model of psoriasis. In vivo studies like these point to itaconate as an important mediator of the resolution of inflammation, potentially providing a novel therapeutic opportunity and furthermore, as an endogenous metabolite there is strong evidence to predict good clinical tolerability.

### Citrate

Citrate is another TCA cycle metabolite that accumulates in activated immune cells. Mitochondrial citrate is converted into isocitrate via cis-aconitate by the enzyme aconitase of the TCA cycle (Fig. 2). Isocitrate dehydrogenase (IDH) will subsequently catalyze the oxidative decarboxylation of isocitrate, producing α-ketoglutarate and CO_2_. Activated macrophages as well as dendritic cells have an increased ratio of isocitrate to αKG and furthermore, reduced expression of *Idh1* which, in combination with increased expression of the citrate carrier (CIC) and ACLY, is thought to contribute to the accumulation of citrate detected.^1,168,190–192^

Accumulated citrate can be exported from the mitochondria via CIC (Fig. 2). A role for CIC in inflammation was first demonstrated when CIC expression levels were upregulated in LPS-activated immune cells, and silencing CIC abrogated production of NO, ROS and prostaglandins.^193^ Cytosolic citrate takes part in a negative feedback loop limiting the rate of glycolysis by directly inhibiting several glycolytic enzymes including phosphofructokinases (PFK) 1 and 2, PDH and SDH but also through limiting availability of the pyruvate kinase substrate fructose-1,6-bisphosphate.^194–196^ Conversion of cytosolic citrate to acetyl-coenzyme A (acetyl-CoA) and oxaloacetate by ACLY is critical for fatty acid synthesis as well as protein acetylation. Acetyl-CoA is further processed into malonyl-CoA by acetyl-CoA carboxylase which can be incorporated into cholesterol or fatty acids, whereas oxaloacetate goes on to become malate, a reaction mediated by malate dehydrogenase (MDH). Cytosolic malate can reenter the mitochondria and subsequently the TCA cycle via CIC (reviewed in ref. ^197^). Thus, these processes have been shown to play an important role in activated macrophages and may provide an attractive target for limiting excessive inflammation.

IDH1 is known to be suppressed in cells activated by pro-inflammatory signals. IL-1β and TNFα suppress activity and expression levels of IDH in primary chondrocytes.^198^ Specific mutations in IDH1 have been linked to several brain cancers including oligodendroglioma, and glioblastoma multiforme. In addition, mutations in IDH1 and IDH2 have been linked to AML.^199^ Using selective IDH inhibitors, such as Enasidenib (AG-221, CC-90007) in patients with mutated IDH, ameliorates production of the oncometabolite (D)-2 hydroxyglutarate (D-2-HG) and associated pro-tumor effects, thereby providing a novel therapeutic approach in cancers such as AML.^200,201^ ACLY is the transferase that catalyzes the conversion of citrate and coenzyme A to acetyl-CoA during fatty acid metabolism. ACLY expression has also been linked to cancer, and targeting or depleting ACLY results in increased production of ROS and induction of apoptosis possibly through a mechanism involving AMPK and p53.^202,203^ ACLY is critical in generating the pool of acetyl CoA that is subsequently used for acetylation of histones.^204–206^ Furthermore, it is citrate-generated acetyl-CoA that is used in acetylation of GAPDH, preventing GAPDH from binding IFNγ mRNA, making ACLY a regulator of this inflammatory cytokine.^165^ Moreover, malonyl CoA, the cofactor required for the PTM malonylation described above, is also generated and regulated by components of the citrate pathway. Hence, the citrate metabolic pathways play central roles in regulating immune responses.

## Concluding remarks

Excessive inflammation is the trademark of many destructive disease states. The current use of drugs with metabolic targets to limit inflammation which might act in part to mimic or promote endogenous anti-inflammatory metabolites, and the growing field of research into immunometabolism provide strong evidence for the therapeutic potential in targeting metabolic processes as a means of controlling immune effector functions and thereby alleviating pathological inflammation. Increasing interest mapping out the roles and mechanisms of metabolites and enzymes in events occurring during the cross-talk between immune cell activation and coupled metabolic processes will no doubt identify targets within metabolic pathways that lie beyond the scope of this review, including amino acid metabolism, one-carbon metabolism, fatty acid oxidation, fatty acid synthesis and others. Much of the data published to date stem from studies using T cells and macrophages, however emerging data identifying metabolic mechanisms and signatures of immune cell populations such as B cells, NK cells and dendritic cells point to new opportunities for therapies in a wide range of indications within autoimmunity, inflammation and cancer. In addition, as we draw comparisons of the similarities between the metabolic properties of cancer cells and activated immune cells, an opportunity to repurpose metabolic cancer therapies for autoimmune disorders arises.

**Fig. 3:**
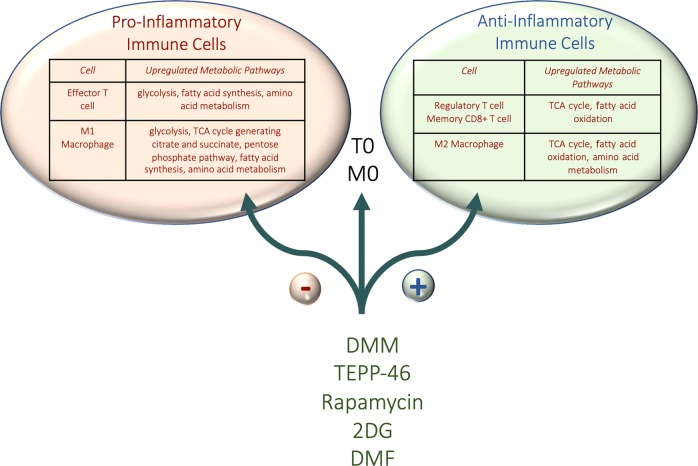
Skewering immune cells towards an anti-inflammatory phenotype using small molecules. By modulating various metabolic pathways of proinflammatory immune cells we can promote a more anti-inflammatory phenotype. Small molecules such as DMM, TEPP-46, Rapamycin, 2DG, DMF and Hyaluronic acid all promote the development of anti-inflammatory Treg cells and M2 macrophages, whilst suppressing differentiation of the more inflammatory Th17 cells and M1 macrophages, which most likely contributes to the efficacious outcomes when using these in models of inflammatory disease.

The prospect of changing the phenotype of an inflammatory cell into an anti-inflammatory one by targeting a specific enzyme in metabolism is tantalizing and may show more success than targeting metabolism in cancer where the aim is to kill the tumor (Fig. 3). Future work in this field is keenly awaited.
